# MicroRNA expression profiles predict clinical phenotypes and prognosis in chromophobe renal cell carcinoma

**DOI:** 10.1038/srep10328

**Published:** 2015-05-18

**Authors:** Yu-Zheng Ge, Hui Xin, Tian-Ze Lu, Zheng Xu, Peng Yu, You-Cai Zhao, Ming-Hao Li, Yan Zhao, Bing Zhong, Xiao Xu, Liu-Hua Zhou, Ran Wu, Lu-Wei Xu, Jian-Ping Wu, Wen-Cheng Li, Jia-Geng Zhu, Rui-Peng Jia

**Affiliations:** 1 Department of Urology, Nanjing First Hospital, Nanjing Medical University, 68 Changle Road, Nanjing 210006, China; 2 Department of Urology, The First Hospital of Nanchang, Nanchang University, 128 Xiangshan North Road, Nanchang 330008, China; 3 Department of Pathology, Nanjing First Hospital, Nanjing Medical University, 68 Changle Road, Nanjing 210006, China; 4 Department of Urology, Xuzhou Third People’s Hospital, Jiangsu University, 131 Huancheng Road, Xuzhou 221005, China; 5 Department of Urology, Huaian First People’s Hospital, Nanjing Medical University, 6 Beijing West Road, Huaian 223300, China; 6 Department of Radiation Oncology, JiangSu Armed Police General Hospital, 8 Jiangdu South Road, Yangzhou 225003, China

## Abstract

Chromophobe renal cell carcinoma (chRCC) is the third most common subtype of kidney cancers. In the present study, we identified 58 treatment-naïve primary chRCC patients from The Cancer Genome Atlas dataset and analyzed the genome-wide microRNA (miRNA) expression profiles, with the aim to assess the relationship of miRNA expression with the progression and prognosis of chRCC. Overall, a total of 105 miRNAs were found to be differentially expressed between tumor and the adjacent normal tissues from 22 chRCC patients. In the unpaired condition (58 chRCC vs. 22 normal tissues), 77 (96.3%) samples were distinguished correctly by the signatures. In the progression-related profiles, 27 miRNAs were selected for pathologic T and 9 for lymph node involvement. In the survival analyses, the expression levels of mir-191, mir-19a, mir-210, and mir-425 were significantly associated with both recurrence-free survival (RFS) and overall survival, while mir-210 was proven as an independent prognostic factor in terms of RFS. In summary, miRNAs are expressed differentially in chRCC, and unique expression of miRNAs is associated with the progression and prognosis of chRCC.

Renal cell carcinoma (RCC) accounts for approximately 3% of all human malignancies, and is one of the most fatal urologic tumors worldwide^1^. In the United States, the newly diagnosed RCC cases and related deaths in 2014 are estimated as 63,920 and 13,860, respectively^2^. Among the various histological subtypes of RCC, chromophobe RCC (chRCC) is the third most prevalent form behind clear cell RCC (ccRCC) and papillary RCC, representing about 5% of all RCC cases^3^,^4^,^5^. Although chRCC typically exhibits an indolent pattern of localized growth with a better prognosis compared to other RCC subtypes, the clinical behavior and long-term outcomes of chRCC are highly variable^6^,^7^,^8^. Therefore, it is of vital importance to identify tumor-specific biomarkers, which could help guide the therapeutic intervention and follow-up strategies^7^.

MicroRNAs (miRNAs) are short (about 19–25 nucleotides in length), non-coding, and single-stranded RNAs that can act as endogenous RNA interference^9^. miRNAs could negatively regulate the expression of hundreds of target genes at the post-translational level, thereby controlling a wide range of biological functions including cellular proliferation, differentiation, and apoptosis^10^. Accumulating evidence has indicated that miRNAs could function as tumor suppressors or carcinogenic factors, and alteration in miRNA expression might exert critical functions in the development and progression of human cancers^11^,^12^,^13^,^14^. The diagnostic and prognostic characteristics of miRNAs have been explored in various cancer types^15^,^16^, and the cancer-specific miRNA expression profiles in ccRCC have been identified, which were significantly associated with patient survival^17^,^18^,^19^. However, the miRNAs in chRCC remain to be elucidated; and the miRNA expression profiles in chRCC and ccRCC vary greatly, which limited the translational application of the findings about ccRCC in the clinical practice of chRCC^20^,^21^.

Hence, we stringently designed a step-wise study using the data from The Cancer Genome Atlas (TCGA) project, which provides a collection of clinicopathological data and the genome-wide miRNA expression profiles^22^. In the current study, we explored the differential expression profiles of miRNAs in chRCC and corresponding normal kidney tissues, and investigated the association between miRNAs and the progression and prognosis of chRCC, with the hope to identify the miRNA expression signatures that could predict the clinical phenotypes and prognosis in chRCC.

## Results

### Patient characteristics

All 58 patients enrolled in the present study were clinically and pathologically diagnosed with chRCC. The median age for all these participants was 49.5 years (inter-quartile range, IQR: 42–62 years), and the median follow-up time was 63.4 months (IQR: 31.5–86.1 months). Overall, eight patients (13.8%) suffered the recurrence after a median follow up of 10.9 months (IQR: 2.4–56.0 months), and seven patients (12.1%) died after a median follow-up time of 25.1 months (IQR: 16.9-38.6 months). Among the 58 participants (Cohort T), twenty-two patients (Cohort M) had obtained the corresponding adjacent normal tissues in addition to the cancerous tissues. As summarized in Table 1, no significant difference was observed in the distribution of age, gender, ethnicity, and American Joint Committee on Cancer (AJCC) tumor-node-metastasis (TNM) information between the two cohorts (all *P* values > 0.05).

### Differentially expressed miRNAs in chRCC vs. adjacent normal tissue

**Figure 1:**
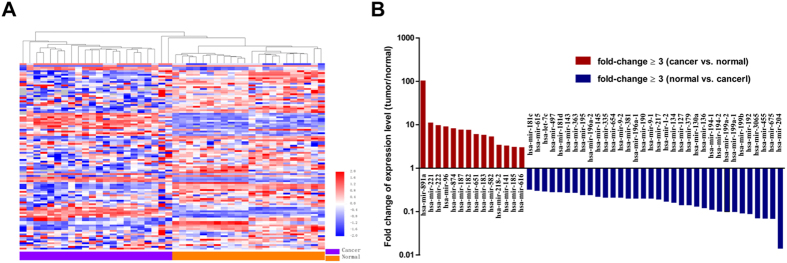
Differentially expressed microRNAs between chromophobe renal cell carcinoma and adjacent normal tissues . **A**. unsupervised hierarchical cluster analysis of the 105 differentially expressed miRNAs between the tumors and matched adjacent normal tissues of 22 patients; **B**. differentially expressed miRNAs with fold-change ≥3.

**Figure 2:**
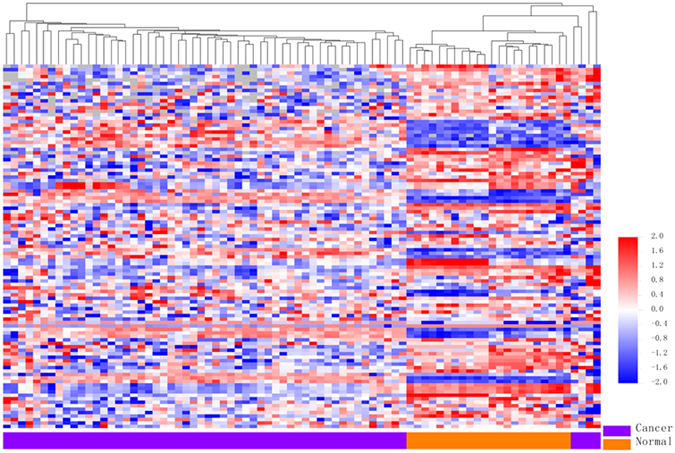
Unsupervised hierarchical cluster analysis of the 105 differentially expressed miRNAs in unpaired conditions.

The miRNA expression in the tumors and matched non-tumor tissues from 22 chRCC patients (Cohort M) was profiled, and a total of 105 miRNAs were found to be expressed differentially after adjustment for multiple testing (Fig. 1 and Supplementary Table S1). Among these 105 miRNAs, 25 miRNAs (23.8%) were up-regulated while the remaining 80 miRNAs (76.2%) were down-regulated. With regard to the fold-change in expression levels, 47 differentially expressed miRNAs showed a greater than 3-fold change in expression levels (Fig. 1B). By class prediction, all samples (22 chRCC vs. 22 matched non-tumor tissues) were classified correctly; even in the unpaired condition (58 chRCC vs. 22 normal tissues), 96.3% (77/80) of samples were classified correctly. Additionally, the unsupervised hierarchical clustering with the 105 miRNAs expression data could clearly separate the tumor and non-tumor samples in both paired (Fig. 1A) and unpaired (Fig. 2) conditions.

### MiRNAs in relation to tumor progression of chRCC

To identify miRNAs associated with tumor progression for each clinical phenotype, the class comparison analyses were conducted. A summary of 27 miRNAs were selected for stage and pathologic T, 9 for lymph node status, and 0 for metastasis status (Supplementary Table S2). Of note, as chRCC is relatively indolent with low incidences of metastasis, and the patients with distant metastasis are rarely recommended to undergo surgery, the number of M1 patients was only two, which could explain the negative result to some extent.

### MiRNA expression profiles associated with chRCC prognosis

**Figure 3:**
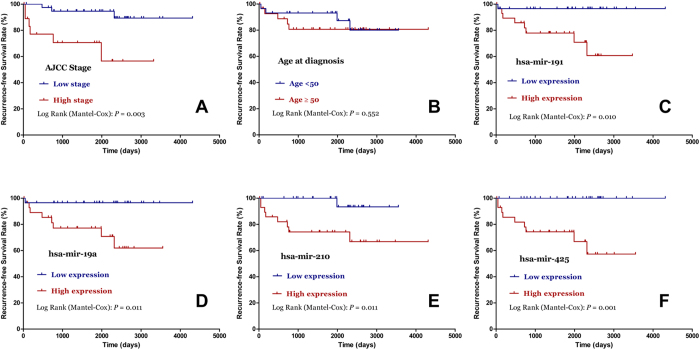
Kaplan–Meier curves for the recurrence-free survival of chromophobe renal cell carcinoma patients. The 58 chromophobe renal cell carcinoma patients were compared in two groups according to: **A**. AJCC stage (stage III+IV vs. stage I+II); **B**. age (cutoff point: 50); **C**. hsa-mir-191; **D**. hsa-mir-19a; **E**. hsa-mir-210; **F**. hsa-mir-425.

**Figure 4:**
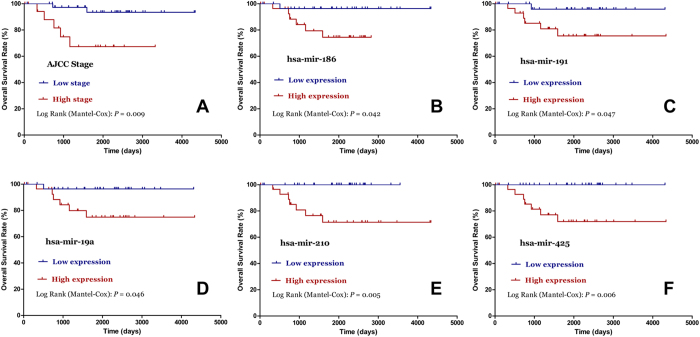
Kaplan–Meier curves for the overall survival of chromophobe renal cell carcinoma patients. The 58 chromophobe renal cell carcinoma patients were compared in two groups according to: **A**. AJCC stage (stage III+IV vs. stage I+II); **B**. hsa-mir-186; **C**. hsa-mir-191; **D**. hsa-mir-19a; **E**. hsa-mir-210; **F**. hsa-mir-425.

As shown in Kaplan-Meier survival analyses (Fig. 3), AJCC stage (*P* = 0.003), mir-191(*P* = 0.010), mir-19a (*P* = 0.011), mir-210 (*P* = 0.011), and mir-425 (*P*=0.001) were significantly associated with recurrence-free survival (RFS) in chRCC patients. Furthermore, the univariate Cox regression analyses indicated that AJCC stage (*P* = 0.010), mir-191 (*P* = 0.035), mir-19a (*P* = 0.036), and mir-210 (*P* = 0.037) were significantly related with the RFS of chRCC patients, and the multivariate Cox regression analyses demonstrated that AJCC stage (*P* = 0.015) and mir-210 (*P* = 0.007) were independent prognostic factors (Table 2). With regard to overall survival (OS) in chRCC patients, stage (*P* = 0.009), mir-186 (*P* = 0.042), mir-191(*P* = 0.047), mir-19a (*P* = 0.046), mir-210 (*P* = 0.005), and mir-425 (*P* = 0.006) were found significantly associated (Fig. 4). However, only AJCCstage was proven as a prognostic factor of OS in the subsequent univariate and multivariate Cox regression analyses.

## Discussion

Aberrant miRNA expression patterns have been documented in various malignancies, and alterations in miRNA expression correlate highly with the progression and prognosis of different cancer types^11^. In the current study, we identified the differentially expressed miRNA profiles between chRCC and normal renal tissues, and the substantial associations of specific miRNAs with the progression and prognosis of chRCC.

As an endogenous family of small, single-stranded, and non-coding RNAs, miRNAs could regulate as much as 30% of the human genome^9^,^23^, and each individual miRNA can regulate the translation of hundreds of target mRNAs^10^,^24^,^25^. Mounting evidence has documented the functions of miRNAs as important regulators in the development and progression of human malignancies^26^,^27^,^28^. To gain more insight into tumor biology, miRNAs profiling has arisen as a major study approach, and widespread dysregulated miRNAs have been demonstrated in various tumor types including ccRCC^29^,^30^. However, the biological functions of miRNAs in the development and progression of chRCC, and the potential diagnostic and prognostic characteristics remain to be elucidated.

In the present study, a summary of 58 treatment-naïve primary chRCC patients were identified from the TCGA project, and enrolled in this genome-wide miRNA expression analysis. One hundred and five miRNAs were found to be differentially expressed between chRCC tumors and matched non-cancerous tissues, which were further validated as robust classifiers even in unpaired conditions. In 2008, Nakada and colleagues compared the genome-wide miRNA expression profiles in 4 chRCC tumors and 6 normal kidney tissues from ccRCC patients, and found 57 differentially expressed miRNAs^31^. Among the 57 miRNAs, twenty were in agreement with the current study, and aberrantly expressed at the same direction (16 down-regulated and 4 up-regulated, chRCC tumors vs. normal tissues; Supplementary Table S3). With regard to the AJCC TNM information, 27 miRNAs were identified for stage and pathologic T, and 9 for lymph node involvement. However, no miRNA was associated with stage, pathologic T, and lymph node involvement. Finally, we investigated the relationship of different miRNA expression levels with the long-term outcomes (RFS and OS), and found four (mir-191, mir-19a, mir-210, and mir-425) and five (mir-186, mir-191, mir-19a, mir-210, and mir-425) miRNAs were significantly related with RFS and OS in chRCC, respectively. After the stepwise univariate and multivariate Cox regression analysis, mi-210 was proven as a potent independent prognostic factor in terms of RFS rather than OS.

Mir-210 is an intronic miRNA located at the telomeric portion of chromosome 11pl5.5^32^. Even though its novel target genes and biological functions remains under discovering, accumulating evidence indicated that mir-210 could exert vital functions in the development and progression of human malignancies, majorly through regulating cancer cell survival, proliferation, differentiation as well as angiogenesis^33^,^34^. In ccRCC, different clinical studies have investigated the prognostic values of mir-210, and found that the high mir-210 expression level in tumor tissue or serum was significantly correlated with poor outcomes^18^,^35^. Our findings were in agreement with the previous studies, and widened the potential utilities of mir-210 in clinical oncology.

Some limitations should be acknowledged in interpreting the results. First, only 1046 miRNAs were initially included in the present study, which accounted for approximately 55.6% of the totally discovered miRNAs in human beings (data from the miRBase)^36^. So, the prognostic miRNAs identified here may not represent all miRNA candidates that are potentially correlated with survival time in chRCC. Second, as the incidence of chRCC is very low with about 3,000 new cases annually in the United States^37^, the number of chRCC patients enrolled in this study was only 58, which might reduce the power to identify more significant miRNAs. Third, the follow-up time (median 63.4 months) in current study was relatively short, as chRCC is an indolent cancer with about 85% five-year survivals^4^. Fourth, even though the chRCC cases were strictly identified according to the predesigned selection criteria and the leave-one-out cross validation method was applied, the false positive results do potentially exist, which call for an external validation cohort to confirm the results.

In summary, by analyzing the genome-wide miRNA expression profiles in an independent chRCC patient cohort, our study identified the miRNAs differentially expressed between cancerous and noncancerous tissues, and those in relation with the progression and prognosis of chRCC. Additionally, further well-designed and unbiased studies with larger sample size and longer follow-up time should be conducted to verify our findings.

## Materials and methods

### Patients and samples

All chRCC patients were identified from the multi-institutional TCGA data portal that underwent partial or radical nephrectomy from 2000 to 2010 for sporadic chRCC^22^. The full clinical data (Level 1 and Level 2) were downloaded (up to July 1, 2014) and double-checked for the further assessment of the eligibility. The subjects that had history of other malignancies and/or received neoadjuvant therapy (chemotherapy or radiation therapy) were excluded. Furthermore, the pathological stage was reevaluated and confirmed by two experienced pathologists according to the 7^th^ edition of TNM classification of AJCC. Overall, a total of 58 chRCC patients were enrolled with full annotation of the corresponding clinical data including age, gender, race, and AJCC TNM information (Table 1). Among the 58 participants (Cohort T), the matched normal tissues (distance from the tumor margin >2 cm) were retrieved from 22 subjects (Cohort M). During the follow-up, the primary end-point was RFS while the secondary end-point was OS. In the TCGA project, all chRCC patients were well informed and provided written consent, and the appropriate approvals were obtained from the institutional review boards at Brigham and Women’s Hospital, MD Anderson Cancer Center, Memorial Sloan-Kettering Cancer Center, and the National Cancer Institute^22^. Furthermore, the protocol of current study was approved by the institutional review board of Nanjing First Hospital, Nanjing Medical University, and the data collection and procession were performed in agreement with the TCGA human subject protection and data access policies.

### Microarray data procession

The summary miRNA data (Level 3) was downloaded from TCGA data portal (up to July 10^th^, 2014). The miRNA expression level was measured with the Illumina HiSeq platform (Illumina Inc, San Diego, CA, USA), quantified by relative miRNA read counts to the total miRNAs read counts and presented as reads per million counts (RPM). The miRNA expression summary data was processed using BRB-Array tools (version 4.4.0; National Cancer Institute, Bethesda, MD, USA) which were developed by Dr. Richard Simon and BRB-Array Tools Development Team^38^. Briefly, the miRNAs were retained when they were more than 1 RPM in at least 10% of all samples and had changes of more than 1.5 fold from the median value in at least 20% of samples. Subsequently, the expression level of each individual miRNA was log2 transformed for further analysis.

### Statistical analysis

The continuous variables were presented as mean ± standard deviation or median (IQR), according to the normality status determined by Kolmogorov-Smirnov and Shapiro-Wilk tests. The differences of clinicopathological variables (gender, race, and AJCC TNM information) between two cohorts (Cohort M and Cohort T) were evaluated using Chi-square test or Fisher exact test, while the difference of age was examined with Student *t* test.

The miRNA expression levels between the two different groups (cancerous vs. matched noncancerous tissues, stage III + IV vs. stage I + II, T3 + T4 vs. T1 + T2, N1 + N2 vs. N0, and M1 vs. M0) were ascertained with paired or unpaired *t*-test (significance level was set as 0.01). In exploring the differential expression profiles of miRNAs in chRCC and non-cancerous tissues, the class prediction with leave-one-out cross validation method was conducted, and hierarchical cluster analysis was performed to generate a tree cluster showing the separation of different classes.

Kaplan-Meier survival and univariate Cox proportional hazards regression analyses were conducted to explore the effects of age, miRNA expression levels (cutoff point: median value), gender, AJCC stage (stage III + IV vs. stage I + II), tumor size (T3+T4 vs. T1+T2), lymph node (N1+N2 vs. N0), and metastasis status (M1 vs. M0) on patient survival (RFS and OS). Furthermore, the multivariate Cox proportional hazards regression analysis was performed by combining the potential prognostic factors (with *P* values <0.10 in the univariate Cox regression analysis). Statistical significance was taken as a two-sided *P* value <0.05 unless specifically indicated. The statistical analyses were performed with the use of BRB-Array Tools, SPSS (version 21.0; SPSS Institute Inc, Chicago, IL, USA), and GraphPad Prism (version 5.0; GraphPad Software, San Diego, CA, USA), as appropriate.

## Author Contributions

Y. Z. G. and R. P. J. conceived and designed the work. H. X., T. Z. L., Z. X., P.  Y., Y. C. Z. and M. H. L. performed the experiments. P. Y., Y. Z., B. Z., X. X., L. H. Z., R. W., L. W. X. and J.P.W. analyzed the data. Y. Z.G, H.X, and T.Z.L wrote the paper. W.C.L, J.G.Z, and R.P.J proofread and revised the paper. R.P.J agreed to be accountable for all aspects of the work. All authors read and approved the final manuscript.

## Additional Information

**How to cite this article**: Ge, Y.-Z. *et al.* MicroRNA expression profiles predict clinical phenotypes and prognosis in chromophobe renal cell carcinoma. *Sci. Rep.* **5**, 10328; doi: 10.1038/srep10328 (2015).

## Supplementary Material

Supplementary Information

## Figures and Tables

**Table 1:** Clinical characteristics of patients with chromophobe renal cell carcinoma

Category	Cohort M (n = 22)	Cohort T (n = 58)	*P* Value
**Age**, Mean ± SD	53.4 ± 13.0	51.3 ± 14.1	0.547
**Race**, n (%)			0.907
Caucasian	20 (90.9%)	52 (89.7%)	
African	1 (4.5%)	4 (6.9%)	
Asian	1 (4.5%)	2 (3.4%)	
**Gender**, n (%)			0.622
Female	11 (50.0%)	25 (43.1%)	
Male	11 (50.0%)	33 (56.9%)	
**AJCC Stage**, n (%)			0.561
Stage I	9 (40.9%)	17 (29.3%)	
Stage II	7 (31.8%)	22 (37.9%)	
Stage III	3 (13.6%)	14 (24.1%)	
Stage IV	3 (13.6%)	5 (8.6%)	
**Tumor size**, n (%)			0.806
T1	9 (40.9%)	17 (29.3%)	
T2	7 (31.8%)	22 (37.9%)	
T3	5 (22.7%)	16 (27.6%)	
T4	1 (4.5%)	3 (5.2%)	
**Lymph node**, n (%)			0.441
N0	11 (50.0%)	38 (65.5%)	
N1+N2	2 (9.1%)	4 (6.9%)	
NX	9 (40.9%)	16 (27.6%)	
**Metastasis status**, n (%)			0.974
M0	18 (81.8%)	48 (82.8%)	
M1	1 (4.5%)	2 (3.4%)	
MX	3 (13.6%)	8 (13.8%)	

N, number of patients; SD, standard deviation; AJCC, American Joint Committee on Cancer; NX, regional lymph node unknown; MX, metastasis status unknown.

**Table 2:** Univariate and multivariate Cox regression analysis of recurrence-free survival in chromophobe renal cell carcinoma patients

MicroRNA	Univariate analysis		Multivariate analysis	
	HR (95%CI)	*P* value	HR (95%CI)	*P* value
Stage (I+II vs. I+II)	6.500 (1.578-26.77)	**0.010**^a^	8.388(1.505-46.74)	**0.015**^a^
hsa-mir-191	9.389 (1.169-75.43)	**0.035**^a^	0.968(0.086-10.89)	0.979
hsa-mir-19a	9.264 (1.157-74.20)	**0.036**^a^	7.057(0.636-78.31)	0.112
hsa-mir-210	9.147 (1.143-73.22)	**0.037**^a^	26.01(2.423-279.1)	**0.007**^a^
hsa-mir-425	84.31 (0.372-19121)	0.109	75528(0.001->1000)	0.958

HR, hazard ratio; 95% CI, 95% confidential interval.

^a^statistical significant results (in bold).
