# Supplementary material for: MicroRNA expression profiles predict clinical phenotypes and prognosis in chromophobe renal cell carcinoma
Source: Sci Rep. 2015 May 18;5:10328. doi: 10.1038/srep10328 (PMC4434887; doi:10.1038/srep10328)
Supplement: Supplementary Information [file srep10328-s1.doc]

**MicroRNA expression profiles predict clinical phenotypes and prognosis in chromophobe renal cell carcinoma**

Yu-Zheng Ge^1^, Hui Xin^1^, Tian-Ze Lu^1^, Zheng Xu^1^, Peng Yu^2^, You-Cai Zhao^3^, Ming-Hao Li^1,3^, Yan Zhao^4^, Bing Zhong^5^, Xiao Xu^6^, Liu-Hua Zhou^1^, Ran Wu^1^, Lu-Wei Xu^1^, Jian-Ping Wu^1^, Wen-Cheng Li^1^, Jia-Geng Zhu^1^, Rui-Peng Jia^1¶^

^1^ Department of Urology, Nanjing First Hospital, Nanjing Medical University, 68 Changle Road, Nanjing 210006, China.

^2^ Department of Urology, The First Hospital of Nanchang, Nanchang University, 128 Xiangshan North Road, Nanchang 330008, China.

^3^ Department of Pathology, Nanjing First Hospital, Nanjing Medical University, 68 Changle Road, Nanjing 210006, China.

^4^ Department of Urology, Xuzhou Third People’s Hospital, Jiangsu University, 131 Huancheng Road, Xuzhou 221005, China.

^5^ Department of Urology, Huaian First People’s Hospital, Nanjing Medical University, 6 Beijing West Road, Huaian 223300, China.

^6^ Department of Radiation Oncology, JiangSu Armed Police General Hospital, 8 Jiangdu South Road, Yangzhou 225003, China

**^¶^ Correspondence author:**

**Rui-Peng Jia**, M.D., Ph.D.,

Professor of Urology, Chief of Department of Urology

Nanjing First Hospital, Nanjing Medical University

68 Changle Road, Nanjing 210006, China.

E-mail: [urojiarp@126.com](mailto:urojiarp@126.com)

Tel: +86-25-52271061

**Legends of supporting information:**

Table S1. **Summary of differentially expressed microRNAs in cancer and adjacent normal tissues**

Table S2. **MicroRNAs associated with the progression of chromophobe renal cell carcinoma**

Table S3. **Summary of the microRNAs identified by both studies**

Table S1. Summary of differentially expressed microRNAs in cancer and adjacent normal tissues

| MicroRNA | Expression Level ^a^ | | Fold Change | *P* value | FDR |
| --- | --- | --- | --- | --- | --- |
|  | chRCC | Normal |  |  |  |
| hsa-mir-891a | 12.64±2.92 | 5.43±1.30 | 104.31 | < 1e-07 | < 1e-07 |
| hsa-mir-221 | 10.70±0.61 | 6.59±0.41 | 11.18 | < 1e-07 | < 1e-07 |
| hsa-mir-222 | 8.97±0.65 | 4.71±0.66 | 9.78 | < 1e-07 | < 1e-07 |
| hsa-mir-96 | 5.44±0.96 | 1.58±0.371 | 9.13 | < 1e-07 | < 1e-07 |
| hsa-mir-874 | 8.55±0.94 | 4.52±0.54 | 8.29 | < 1e-07 | < 1e-07 |
| hsa-mir-187 | 7.51±2.41 | 3.63±1.82 | 7.72 | 3.76E-05 | 0.000109 |
| hsa-mir-182 | 14.54±1.48 | 11.04±1.16 | 7.67 | < 1e-07 | < 1e-07 |
| hsa-mir-651 | 4.12±1.60 | 0.99±0.57 | 6.11 | 4.00E-07 | 1.89E-06 |
| hsa-mir-183 | 12.67±1.33 | 9.49±1.11 | 5.86 | < 1e-07 | 5.29E-07 |
| hsa-mir-582 | 10.32±0.71 | 7.07±0.41 | 5.36 | < 1e-07 | < 1e-07 |
| hsa-mir-218-2 | 7.45±2.00 | 4.87±0.95 | 3.44 | 0.0008449 | 0.00175 |
| hsa-mir-141 | 10.20±1.97 | 7.72±0.70 | 3.31 | 0.000307 | 0.000716 |
| hsa-mir-185 | 7.48±0.75 | 4.90±0.62 | 3.08 | < 1e-07 | < 1e-07 |
| hsa-mir-616 | 3.13±0.83 | 0.81±0.33 | 3.03 | < 1e-07 | < 1e-07 |
| hsa-mir-942 | 2.76±0.87 | 0.80±0.345 | 2.75 | 9.00E-07 | 3.83E-06 |
| hsa-mir-766 | 4.22±0.91 | 2.20±0.60 | 2.36 | 5.30E-06 | 1.95E-05 |
| hsa-mir-576 | 3.62±0.76 | 1.68±0.40 | 2.24 | 5.00E-07 | 2.26E-06 |
| hsa-mir-3653 | 3.23±0.91 | 1.42±0.58 | 2.12 | 5.70E-06 | 2.06E-05 |
| hsa-mir-671 | 3.27±0.64 | 1.46±0.33 | 1.99 | 5.80E-06 | 2.06E-05 |
| hsa-mir-22 | 17.39±0.38 | 16.23±0.44 | 1.97 | 3.10E-06 | 1.27E-05 |
| hsa-mir-598 | 5.89±0.97 | 4.02±0.38 | 1.97 | 2.34E-05 | 7.25E-05 |
| hsa-mir-3607 | 5.87±0.94 | 4.00±0.73 | 1.9 | 0.000318 | 0.000723 |
| hsa-mir-589 | 6.58±0.60 | 4.89±0.72 | 1.77 | 9.94E-05 | 0.000251 |
| hsa-mir-2355 | 5.27±0.87 | 3.73±0.38 | 1.6 | 0.0009446 | 0.00193 |
| hsa-mir-340 | 4.14±0.50 | 2.71±0.36 | 1.47 | 0.0004004 | 0.000887 |
| hsa-mir-128-2 | 5.58±0.37 | 5.41±0.39 | 0.71 | 0.0006713 | 0.00143 |
| hsa-mir-423 | 6.62±0.42 | 6.33±0.32 | 0.7 | 0.0007425 | 0.00155 |
| hsa-mir-99b | 14.84±0.62 | 14.95±0.24 | 0.67 | 0.000188 | 0.000454 |
| hsa-mir-101-1 | 14.12±0.44 | 14.29±0.35 | 0.65 | 0.000108 | 0.000269 |
| hsa-mir-128-1 | 6.03±0.43 | 5.92±0.38 | 0.65 | 1.94E-05 | 6.44E-05 |
| hsa-mir-101-2 | 5.42±0.54 | 5.29±0.48 | 0.64 | 0.0002698 | 0.000636 |
| hsa-let-7a-1 | 12.22±0.31 | 12.64±0.30 | 0.62 | 3.00E-07 | 1.45E-06 |
| hsa-let-7a-2 | 13.40±0.40 | 13.84±0.37 | 0.61 | 3.00E-07 | 1.45E-06 |
| hsa-mir-103-1 | 12.85±0.68 | 13.25±0.28 | 0.6 | 2.48E-05 | 7.58E-05 |
| hsa-mir-744 | 4.62±0.50 | 4.46±0.35 | 0.6 | 0.0003197 | 0.000723 |
| hsa-let-7a-3 | 12.29±0.31 | 12.87±0.22 | 0.59 | 2.00E-07 | 1.03E-06 |
| hsa-mir-24-2 | 9.81±0.53 | 9.80±0.29 | 0.59 | 4.59E-05 | 0.000126 |
| hsa-mir-30a | 16.78±0.38 | 17.52±0.21 | 0.58 | 6.00E-07 | 2.60E-06 |
| hsa-mir-345 | 2.90±0.95 | 2.70±0.61 | 0.58 | 0.0007295 | 0.00154 |
| hsa-mir-30c-2 | 10.63±0.30 | 10.73±0.58 | 0.57 | 2.07E-05 | 6.61E-05 |
| hsa-mir-98 | 5.25±0.44 | 5.29±0.37 | 0.57 | 4.04E-05 | 0.000114 |
| hsa-mir-126 | 11.69±0.60 | 12.33±0.31 | 0.54 | 6.30E-06 | 2.17E-05 |
| hsa-mir-320b-2 | 1.13±0.51 | 1.41±0.52 | 0.54 | 5.25E-05 | 0.000142 |
| hsa-mir-19b-2 | 5.26±0.53 | 5.41±0.44 | 0.53 | 6.22E-05 | 0.000163 |
| hsa-mir-152 | 7.73±0.55 | 7.67±0.32 | 0.52 | 4.10E-06 | 1.56E-05 |
| hsa-mir-27a | 9.24±0.78 | 9.25±0.32 | 0.52 | 5.78E-05 | 0.000153 |
| hsa-mir-450a-2 | 2.29±0.77 | 2.38±0.43 | 0.52 | 0.0004797 | 0.00104 |
| hsa-mir-181a-1 | 10.23±0.50 | 10.49±0.40 | 0.51 | 0.0003545 | 2.26E-06 |
| hsa-mir-193a | 7.03±0.98 | 7.03±0.62 | 0.51 | 5.00E-07 | 0.00108 |
| hsa-mir-505 | 4.80±0.52 | 5.00±0.31 | 0.51 | 0.0005005 | 0.000109 |
| hsa-mir-139 | 7.07±1.06 | 7.17±0.26 | 0.48 | 3.80E-05 | 0.000153 |
| hsa-mir-181b-1 | 7.61±0.85 | 7.67±0.35 | 0.48 | 5.72E-05 | 1.36E-05 |
| hsa-mir-29c | 10.93±0.57 | 11.62±0.83 | 0.48 | 3.50E-06 | 7.08E-05 |
| hsa-mir-326 | 1.43±0.64 | 1.88±0.52 | 0.48 | 2.25E-05 | 0.000108 |
| hsa-mir-338 | 7.23±0.69 | 7.35±0.74 | 0.46 | 3.70E-05 | 9.92E-05 |
| hsa-mir-99a | 9.67±0.95 | 9.89±0.42 | 0.46 | 3.29E-05 | 0.000177 |
| hsa-mir-132 | 6.16±0.91 | 6.44±0.91 | 0.45 | 6.87E-05 | 0.000454 |
| hsa-mir-146b | 6.71±1.07 | 6.93±0.80 | 0.45 | 0.0001885 | 0.000107 |
| hsa-mir-212 | 1.71±0.86 | 2.12±0.73 | 0.45 | 3.59E-05 | 0.00104 |
| hsa-mir-23b | 10.09±0.59 | 10.61±0.353 | 0.43 | 0.0004815 | 5.29E-07 |
| hsa-mir-301a | 2.00±0.82 | 2.34±0.34 | 0.43 | 1.00E-07 | 2.61E-05 |
| hsa-let-7b | 13.03±0.65 | 13.97±0.38 | 0.42 | 7.70E-06 | 5.29E-07 |
| hsa-mir-106a | 3.35±1.02 | 3.68±0.60 | 0.42 | 1.00E-07 | 0.000348 |
| hsa-mir-1180 | 3.78±0.78 | 4.10±0.55 | 0.42 | 0.0001411 | 1.34E-05 |
| hsa-mir-24-1 | 4.19±0.80 | 4.64±0.48 | 0.41 | 3.40E-06 | 1.21E-05 |
| hsa-mir-542 | 8.28±0.87 | 8.46±0.36 | 0.41 | 2.90E-06 | 2.17E-05 |
| hsa-mir-149 | 2.76±1.04 | 3.26±0.62 | 0.38 | 6.20E-06 | 0.000114 |
| hsa-mir-26b | 8.19±0.39 | 8.52±0.46 | 0.38 | 4.11E-05 | < 1e-07 |
| hsa-mir-27b | 10.25±0.45 | 10.97±0.45 | 0.37 | < 1e-07 | < 1e-07 |
| hsa-mir-1287 | 3.62±0.55 | 4.03±0.42 | 0.36 | < 1e-07 | < 1e-07 |
| hsa-mir-409 | 1.97±0.99 | 2.63±0.70 | 0.34 | < 1e-07 | 6.61E-05 |
| hsa-mir-181c | 4.93±0.62 | 5.81±0.64 | 0.32 | 2.07E-05 | < 1e-07 |
| hsa-mir-615 | 2.26±1.17 | 3.07±0.42 | 0.3 | < 1e-07 | 1.76E-05 |
| hsa-let-7c | 10.33±0.70 | 11.52±0.58 | 0.29 | 4.70E-06 | < 1e-07 |
| hsa-mir-181d | 2.31±0.95 | 3.26±0.76 | 0.28 | < 1e-07 | < 1e-07 |
| hsa-mir-497 | 3.25±0.91 | 4.15±0.66 | 0.28 | < 1e-07 | < 1e-07 |
| hsa-mir-143 | 14.69±0.82 | 16.16±0.58 | 0.27 | < 1e-07 | < 1e-07 |
| hsa-mir-363 | 3.45±1.43 | 4.43±0.56 | 0.27 | < 1e-07 | 6.44E-05 |
| hsa-mir-195 | 3.95±0.70 | 5.09±0.65 | 0.24 | 1.96E-05 | < 1e-07 |
| hsa-mir-196a-2 | 3.85±1.32 | 5.12±0.57 | 0.24 | < 1e-07 | 1.45E-06 |
| hsa-mir-145 | 9.33±0.98 | 10.73±0.90 | 0.22 | 3.00E-07 | 5.29E-07 |
| hsa-mir-335 | 3.74±1.13 | 5.04±0.51 | 0.21 | < 1e-07 | < 1e-07 |
| hsa-mir-654 | 1.64±1.23 | 2.85±0.78 | 0.21 | < 1e-07 | 1.33E-05 |
| hsa-mir-9-2 | 7.30±2.75 | 8.80±1.22 | 0.21 | 3.30E-06 | 0.000723 |
| hsa-mir-190 | 2.21±1.62 | 3.66±0.78 | 0.2 | 0.0003136 | 2.60E-06 |
| hsa-mir-196a-1 | 7.02±1.42 | 8.41±0.50 | 0.2 | 6.00E-07 | < 1e-07 |
| hsa-mir-381 | 2.35±0.92 | 3.78±0.58 | 0.2 | < 1e-07 | < 1e-07 |
| hsa-mir-9-1 | 7.27±2.75 | 8.86±1.30 | 0.2 | < 1e-07 | 0.000473 |
| hsa-mir-217 | 3.00±1.50 | 4.63±2.19 | 0.19 | 0.0001985 | 0.000213 |
| hsa-mir-1-2 | 1.82±1.09 | 3.56±0.97 | 0.17 | 8.33E-05 | < 1e-07 |
| hsa-mir-134 | 4.41±1.10 | 6.22±0.71 | 0.16 | < 1e-07 | < 1e-07 |
| hsa-mir-127 | 6.29±1.14 | 8.23±0.71 | 0.14 | < 1e-07 | < 1e-07 |
| hsa-mir-379 | 5.69±1.12 | 7.63±0.73 | 0.14 | < 1e-07 | < 1e-07 |
| hsa-mir-130a | 4.36±0.85 | 6.45±0.28 | 0.13 | < 1e-07 | < 1e-07 |
| hsa-mir-136 | 1.98±1.07 | 4.19±0.63 | 0.12 | < 1e-07 | < 1e-07 |
| hsa-mir-194-1 | 6.62±0.90 | 8.88±1.84 | 0.11 | < 1e-07 | < 1e-07 |
| hsa-mir-194-2 | 6.77±0.93 | 9.20±1.86 | 0.1 | < 1e-07 | < 1e-07 |
| hsa-mir-199a-2 | 7.95±1.11 | 10.46±0.46 | 0.099 | < 1e-07 | < 1e-07 |
| hsa-mir-199a-1 | 7.17±1.16 | 9.62±0.45 | 0.098 | < 1e-07 | < 1e-07 |
| hsa-mir-199b | 8.46±1.08 | 11.06±0.55 | 0.091 | < 1e-07 | < 1e-07 |
| hsa-mir-192 | 9.17±0.90 | 12.02±2.18 | 0.088 | < 1e-07 | < 1e-07 |
| hsa-mir-3065 | 2.32±1.42 | 5.39±1.16 | 0.07 | < 1e-07 | < 1e-07 |
| hsa-mir-455 | 5.37±1.28 | 8.26±0.37 | 0.069 | < 1e-07 | < 1e-07 |
| hsa-mir-675 | 2.70±1.35 | 5.71±1.96 | 0.068 | < 1e-07 | 5.29E-07 |
| hsa-mir-204 | 3.48±2.57 | 8.79±1.13 | 0.014 | < 1e-07 | < 1e-07 |

chRCC, chromophobe renal cell carcinoma; FDR, false discovery rate

^a^ log2 transformed expression data

Table S2. MicroRNAs associated with the progression of chromophobe renal cell carcinoma

| MicroRNA | Expression Level ^a^ | | Fold Change | *P* value | FDR |
| --- | --- | --- | --- | --- | --- |
| **AJCC stage^b^** | **High stage** | **Low stage** |  |  |  |
| hsa-mir-127 | 7.19±2.27 | 5.78±1.19 | 2.66 | 0.0022742 | 0.0338 |
| hsa-mir-129-1 | 4.07±1.73 | 2.58±1.37 | 2.81 | 0.0007112 | 0.029 |
| hsa-mir-129-2 | 4.00±1.81 | 2.64±1.19 | 2.56 | 0.0012543 | 0.029 |
| hsa-mir-134 | 4.88±2.49 | 3.57±1.24 | 2.49 | 0.0074494 | 0.0596 |
| hsa-mir-136 | 2.83±1.98 | 1.39±1.00 | 2.7 | 0.0006675 | 0.029 |
| hsa-mir-141 | 8.21±3.15 | 10.32±1.72 | 0.23 | 0.0012332 | 0.029 |
| hsa-mir-152 | 7.52±0.91 | 6.90±0.70 | 1.53 | 0.005724 | 0.0596 |
| hsa-mir-181a-1 | 10.24±0.73 | 9.54±0.62 | 1.62 | 0.0003818 | 0.029 |
| hsa-mir-181b-1 | 7.62±0.83 | 6.85±0.76 | 1.7 | 0.0008674 | 0.029 |
| hsa-mir-185 | 7.23±0.51 | 6.75±0.60 | 1.4 | 0.0048958 | 0.0596 |
| hsa-mir-199a-1 | 7.70±1.85 | 6.33±1.09 | 2.57 | 0.0006534 | 0.029 |
| hsa-mir-199a-2 | 8.42±1.98 | 7.14±1.06 | 2.43 | 0.0016963 | 0.0291 |
| hsa-mir-199b | 8.78±1.95 | 7.54±1.03 | 2.37 | 0.0018212 | 0.0291 |
| hsa-mir-200c | 9.82±3.08 | 11.89±1.83 | 0.24 | 0.001614 | 0.0291 |
| hsa-mir-21 | 15.51±1.30 | 14.56±0.87 | 1.92 | 0.0014746 | 0.0291 |
| hsa-mir-223 | 5.47±1.73 | 4.49±0.95 | 1.97 | 0.0059194 | 0.0596 |
| hsa-mir-24-2 | 9.68±0.75 | 9.23±0.45 | 1.36 | 0.0072143 | 0.0596 |
| hsa-mir-25 | 11.94±0.67 | 11.49±0.49 | 1.37 | 0.0065383 | 0.0596 |
| hsa-mir-324 | 4.97±0.62 | 4.48±0.51 | 1.41 | 0.0027868 | 0.0386 |
| hsa-mir-338 | 5.59±0.52 | 6.55±0.72 | 1.66 | 0.0010818 | 0.029 |
| hsa-mir-340 | 3.141±0.90 | 3.13±0.66 | 1.45 | 0.0055678 | 0.0596 |
| hsa-mir-379 | 6.45±2.58 | 5.12±1.15 | 2.51 | 0.0071481 | 0.0596 |
| hsa-mir-381 | 3.06±2.21 | 1.88±1.09 | 2.27 | 0.0089545 | 0.069 |
| hsa-mir-425 | 6.09±0.74 | 5.57±0.63 | 1.44 | 0.0070501 | 0.0596 |
| hsa-mir-769 | 3.83±0.58 | 3.37±0.53 | 1.37 | 0.0051988 | 0.0596 |
| hsa-mir-92b | 6.01±0.65 | 5.39±0.62 | 1.54 | 0.000958 | 0.029 |
| hsa-mir-93 | 11.31±0.74 | 10.83±0.51 | 1.4 | 0.0060411 | 0.0596 |
| **Lymph node** | **N1+N2** | **N0** |  |  |  |
| hsa-mir-130b | 3.57±2.09 | 2.03±0.85 | 2.91 | 0.0043947 | 0.191 |
| hsa-mir-146b | 7.51±1.63 | 6.18±0.88 | 2.51 | 0.0099088 | 0.191 |
| hsa-mir-16-2 | 3.54±1.47 | 2.15±0.94 | 2.64 | 0.0092193 | 0.191 |
| hsa-mir-181a-2 | 10.30±0.80 | 8.76±1.00 | 2.91 | 0.0042265 | 0.191 |
| hsa-mir-210 | 9.53±1.52 | 7.51±1.30 | 4.05 | 0.0045492 | 0.191 |
| hsa-mir-29b-1 | 9.87±1.22 | 8.89±0.48 | 1.97 | 0.0028324 | 0.191 |
| hsa-mir-29b-2 | 9.85±1.13 | 8.96±0.45 | 1.86 | 0.0035015 | 0.191 |
| hsa-mir-505 | 4.91±1.41 | 3.88±0.64 | 2.05 | 0.0091067 | 0.191 |
| hsa-mir-891a | 10.96±3.70 | 13.69±1.67 | 0.15 | 0.006709 | 0.191 |

FDR, false discovery rate

^a^ log2 transformed expression data

^b^ the miRNAs selected for stage and pathologic T were the same ones

Table S3. Summary of the microRNAs identified by both studies

| MicroRNA | Fold Change ^a^ | |
| --- | --- | --- |
|  | Nakada study ^b^ | Our study |
| **Down-regulated microRNAs** | | |
| hsa-mir-127 | 0.03 | 0.14 |
| hsa-mir-130a | 0.11 | 0.13 |
| hsa-mir-136 | 0.05 | 0.12 |
| hsa-mir-146b | 0.19 | 0.45 |
| hsa-mir-192 | 0.02 | 0.088 |
| hsa-mir-194-1 | 0.02 | 0.11 |
| hsa-mir-196a-1 | 0.15 | 0.2 |
| hsa-mir-204 | 0.01 | 0.014 |
| hsa-mir-326 | 0.05 | 0.48 |
| hsa-mir-335 | 0.1 | 0.21 |
| hsa-mir-363 | 0.07 | 0.27 |
| hsa-mir-379 | 0.05 | 0.14 |
| hsa-mir-381 | 0.02 | 0.2 |
| hsa-mir-455 | 0.02 | 0.069 |
| hsa-mir-497 | 0.14 | 0.28 |
| hsa-mir-615 | 0.04 | 0.3 |
| **Up-regulated microRNAs** | | |
| hsa-mir-182 | 11.19 | 7.67 |
| hsa-mir-183 | 7.47 | 5.86 |
| hsa-mir-222 | 11.28 | 9.78 |
| hsa-mir-96 | 202.68 | 9.13 |

^a^ The fold change of the express levels between tumors versus normal kidney tissues;

^b^ The data was extracted from the study: Nakada, C. et al. Genome-wide microRNA expression profiling in renal cell carcinoma: significant down-regulation of miR-141 and miR-200c. J Pathol 216, 418-427, doi:10.1002/path.2437 (2008).
